# Standardised (plain) cigarette packaging increases attention to both text-based and graphical health warnings: experimental evidence

**DOI:** 10.1016/j.puhe.2014.10.019

**Published:** 2015-01

**Authors:** M. Shankleman, C. Sykes, K.L. Mandeville, S. Di Costa, K. Yarrow

**Affiliations:** a Department of Psychology, City University London, London, UK; b Barts Health NHS Trust, London, UK; c Department of Global Health & Development, London School of Hygiene & Tropical Medicine, London, UK; d Institute of Cognitive Neuroscience & Department of Psychology, University College London, London, UK

**Keywords:** Cigarettes, Standardised packaging, Health warnings, Eye tracking, Attention, UK

## Abstract

**Objective:**

To investigate whether standardised cigarette packaging increases the time spent looking at health warnings, regardless of the format of those warnings.

**Study design:**

A factorial (two pack styles x three warning types) within-subject experiment, with participants randomised to different orders of conditions, completed at a university in London, UK.

**Methods:**

Mock-ups of cigarette packets were presented to participants with their branded portion in either standardised (plain) or manufacturer-designed (branded) format. Health warnings were present on all packets, representing all three types currently in use in the UK: black & white text, colour text, or colour images with accompanying text. Gaze position was recorded using a specialised eye tracker, providing the main outcome measure, which was the mean proportion of a five-second viewing period spent gazing at the warning-label region of the packet.

**Results:**

An opportunity sample of 30 (six male, mean age = 23) young adults met the following inclusion criteria: 1) not currently a smoker; 2) <100 lifetime cigarettes smoked; 3) gaze position successfully tracked for > 50% viewing time. These participants spent a greater proportion of the available time gazing at the warning-label region when the branded section of the pack was standardised (following current Australian guidelines) rather than containing the manufacturer's preferred design (mean difference in proportions = 0.078, 95% confidence interval 0.049 to 0.106, *p* < 0.001). There was no evidence that this effect varied based on the type of warning label (black & white text vs. colour text vs. colour image & text; interaction *p* = 0.295).

**Conclusions:**

During incidental viewing of cigarette packets, young adult never-smokers are likely to spend more time looking at health warnings if manufacturers are compelled to use standardised packaging, regardless of the warning design.

## Highlights

• Standardised cigarette packs could increase the saliency of health warnings.
• Eye tracking can reveal how visual attention is allocated.
• Previous studies only assessed combined image-and-text health warnings.
• We found standardised packs increased attention to *all* current UK warning types.
• This provides additional support for statutory standardised cigarette packaging.

## Introduction

Tobacco use is a global public-health priority. Half of users will die prematurely because of their habit.^1^ In England, cigarette smoking is responsible for an estimated 17% of all deaths of adults aged 35 and over, i.e. around. 80,000 people a year.^2^ Once smokers start, it is very hard to give up (two thirds of smokers would like to quit) and young people are particularly vulnerable, with the majority of smokers starting before the age of 18.^2^ Various tobacco control measures have been proposed to help reduce the number of new smokers taking up the habit, including the use of standardised packaging for tobacco products. Standardised packaging (also known as plain packaging, although this term may be less restrictive) requires that all logos, graphics, and colours be removed, leaving only the brand name of the tobacco company in a simple, standard font against a neutrally coloured pack.^3^ These measures became a legal requirement in Australia in 2012, and the governments of New Zealand, the Republic of Ireland and France have since committed to adopting standardised packaging. The UK government, having initially appeared to reject standardised packaging, is now developing regulations for its introduction.^4^ There are several potential benefits to standardised packaging, which include decreasing the appeal of cigarette packs, reducing confusion between different colour packs and associated health risks, and potentially increasing the effectiveness of health warnings.^5^ Indeed, a systematic review commissioned for the UK government's first consultation suggested that standardised packaging enhances the salience of health warnings.^6^ The review identified a number of studies investigating, for example, the effect of standardised packaging on both recall and assessment of health warnings. It is noteworthy, however, that the review identified only one study recording *eye movements*, which are arguably the most direct and objective measure available for investigating visual attention.^7^

Because the fovea (central retina) of the eye is much more sensitive than the peripheral retina, visual acuity falls off very rapidly with distance from the current point of gaze, which is known as ‘fixation.’ For example, a letter positioned just 2° of visual angle from fixation must be around twice as large as a letter positioned 1° from fixation to be equally readable.^8^ In other words, people mainly see the things at which they are looking directly. Consequently, regular and rapid (saccadic) eye movements are made in order to fixate (i.e. look directly at) key locations within an image.^9^ Hence fixating on health warnings can be considered a prerequisite for any effect on smoking behaviour. Cigarette packs are typically retained after purchase and displayed during use. In these situations, smokers and their non-smoking peers are likely to glance at the manufacturer's branding and/or health warnings. Branding is of course designed to be ‘eye-catching’, by taking advantage of the eyes' tendency to automatically seek areas of high image contrast.^10^ Consistent with this, two studies now show that the removal of manufacturer-designed branding increases the number of saccades towards health warnings, at least for some categories of smokers and non-smokers, in both adults and adolescents.^7,11^ A further pilot study has suggested that standardised packaging can also increase gaze times on warning labels, at least early on during a simulated cigarette selection task.^12^

Importantly, previous full-length reports have chosen to investigate image-based warning labels, which have been found to be more effective as health messages than small, text-only warnings.^13^ However, these are used only on the back of packs in the UK, where front-of-pack warnings are currently black & white text only. Black & white text may interact with image branding in different ways to colour images and/or text when generating automatic cues for eye movements.^10^ Therefore, it is important to determine how a move to standardised packaging might affect the time spent viewing different categories of health warnings, in order to inform warning-label selection under any new regulations. Here, an experiment comparing the effects of standardised packaging on viewing time for black & white text-only, colour text-only, and colour image-and-text warning labels is reported. The authors hypothesized that, regardless of general variations in the amount of time spent fixating the different kinds of warning, standardized packaging would always increase viewing time relative to branded packaging. A sample of relatively young never-smokers was sought for two reasons: (i) policy debates around standardized packaging focus particularly on its potential to prevent initiation of smoking in young people, and (ii) recent eye-tracking evidence suggests that regular smokers actively avoid looking at cigarette packet health warnings regardless of packaging style.^7,11,14^ A different task and analysis relative to previous reports was also used, in order to improve the collective generalizability of eye-tracking studies investigating attention to health warnings.

## Methods

### Design

This study used a factorial (2 × 3) within-subjects design. Two factors were varied: packaging style and type of health warning. Packaging style had two levels: *branded* vs *standardized* packs. The health warning types, with three levels, varied between monochrome text-only warnings (*black & white text*), graphic colour warnings containing an image alongside a text warning (*colour image & text*), and colour text-only warnings (*colour text*). Two representative examples (exemplars) from each of these three categories of health warning were selected for inclusion in the experiment from those currently in use in the UK, based on a pilot study (see Materials, below). Therefore, each participant viewed a total of 12 cigarette packs (two packaging styles × three warning types × two exemplars) with the order of presentation randomized for each participant to mitigate order effects. The main outcome measure was the proportion of time spent gazing at the warning label region of interest (bottom 40% of the pack), but the proportion of time spent gazing at the branding region of interest (top 60% of the pack) is also reported.

### Participants

32 participants were recruited through opportunity sampling at City University London. Two participants completed the experiment but were excluded from further analysis due to technical problems during eye tracking (*n* = 1; no eye position recoverable for > 50% of viewing time) or having smoked more than 100 cigarettes in their lifetime (*n* = 1). The 30 remaining participants (six males and 24 females aged between 19 and 40, mean age = 23, SD = 4.4) defined themselves as not currently smoking and having smoked less than 100 cigarettes in their lifetime, i.e. ‘never-smokers’.^15^ Most were full-time students (*n* = 26, 87%). All participants were English speaking and reported having normal or corrected-to-normal vision. Sample size was determined by pre-specifying a recruitment period ending in August 2013, with the additional requirement that the final sample should be equal to or greater than 24 (in order to be comparable with previous studies on this topic that achieved significant results) and a multiple of 6 (for counterbalancing purposes; see Materials, below). Testing took place individually in a dedicated laboratory, following informed consent procedures.

### Apparatus & primary outcome measure

Visual stimuli (cigarette packs) were presented individually against a white background using a PC running Eprime Version 2.0 (Psychology Software Tools Inc., Sharpsburg, USA). Each pack was centred on a 23-inch LCD monitor refreshing at 60 Hz. The viewing distance was approximately 60 cm from the screen, with each packet subtending ∼7.5 × 11.5° visual angle. A model TX300 video eye tracker (Tobii Technology AB, Danderyd, Sweden) recorded eye gaze data from both eyes simultaneously at 120 Hz (i.e. 8.3 ms per sample). The default nine-point calibration procedure was used to calibrate the eye tracker. The Eprime software controlling image presentation was interfaced with the eye tracker, which permitted the synchronisation of eye gaze data with timing of screen events, and the identification of fixations falling within predefined regions of interest (Eprime extensions for Tobii; Psychology Software Tools Inc., Sharpsburg, USA). Specifically, a sample-by-sample eye-position data file was created during the stimulus-on period of each trial. This file flagged whenever the eyes where in the branding region of interest or the warning-label region of interest alongside additional eye metrics, and also recorded details of the current experimental condition.

For the pre-analysis, eye-position files were imported into Matlab R2011 (The MathWorks Inc., Natick, USA) where an automated script recovered the accumulated viewing time in each region of interest. The script summarized these data at the participant level as the mean proportion of total stimulus time spent in each region of interest in each experimental condition, with the primary outcome measure being the proportion of gaze time spent in the *warning label* region.

### Intervention

Participants were told that the aim of the study was to examine attitudes towards cigarette packaging. After completing a short demographic questionnaire (date of birth; gender; employment; living arrangements; education; smoking status), they were seated before the eye tracker. Following successful calibration (i.e. eye positions within the corresponding fixation circle for each point as indicated by the eye tracker's automated display), participants received on-screen instructions and were encouraged to ask any additional questions before commencing the experimental procedure.

To begin each trial, participants focussed on a central fixation cross for 2 s to ensure a constant gaze position at image onset. Gaze was then monitored during the presentation of a cigarette packet, which lasted for a fixed period of 5 s per trial. Each presentation was followed by an on-screen question asking participants to rate how appealing they found the packaging using a ten-point Likert scale. These judgments were not analysed, with the task designed to encourage participants to examine the packets through a seemingly purposeful activity whilst diverting mental focus from the eye tracking.

## Materials

The visual stimuli were identically sized branded or standardized cigarette packages. The branded cigarette packages were scanned copies of six popular brands currently available in the United Kingdom: Benson & Hedges, Camel, Lambert & Butler, Lucky Strike, Pall Mall, and Richmond. The appearance of the standardized pack images was based on the current Australian guidelines: the colour selected was Pantone 448C, and a white Helvetica typeface was used to denote the brand and brand variation.^16^ In the UK, warnings currently appear at the bottom of the pack, and differ in size between front and back. The authors opted to standardize all warnings to 40% of the pack size, approximating European regulations at the time of testing.^17^ The packs were created using Adobe Photoshop CS5, and exported as two separate (branding and warning) regions in .jpg format.

**Fig. 1:**
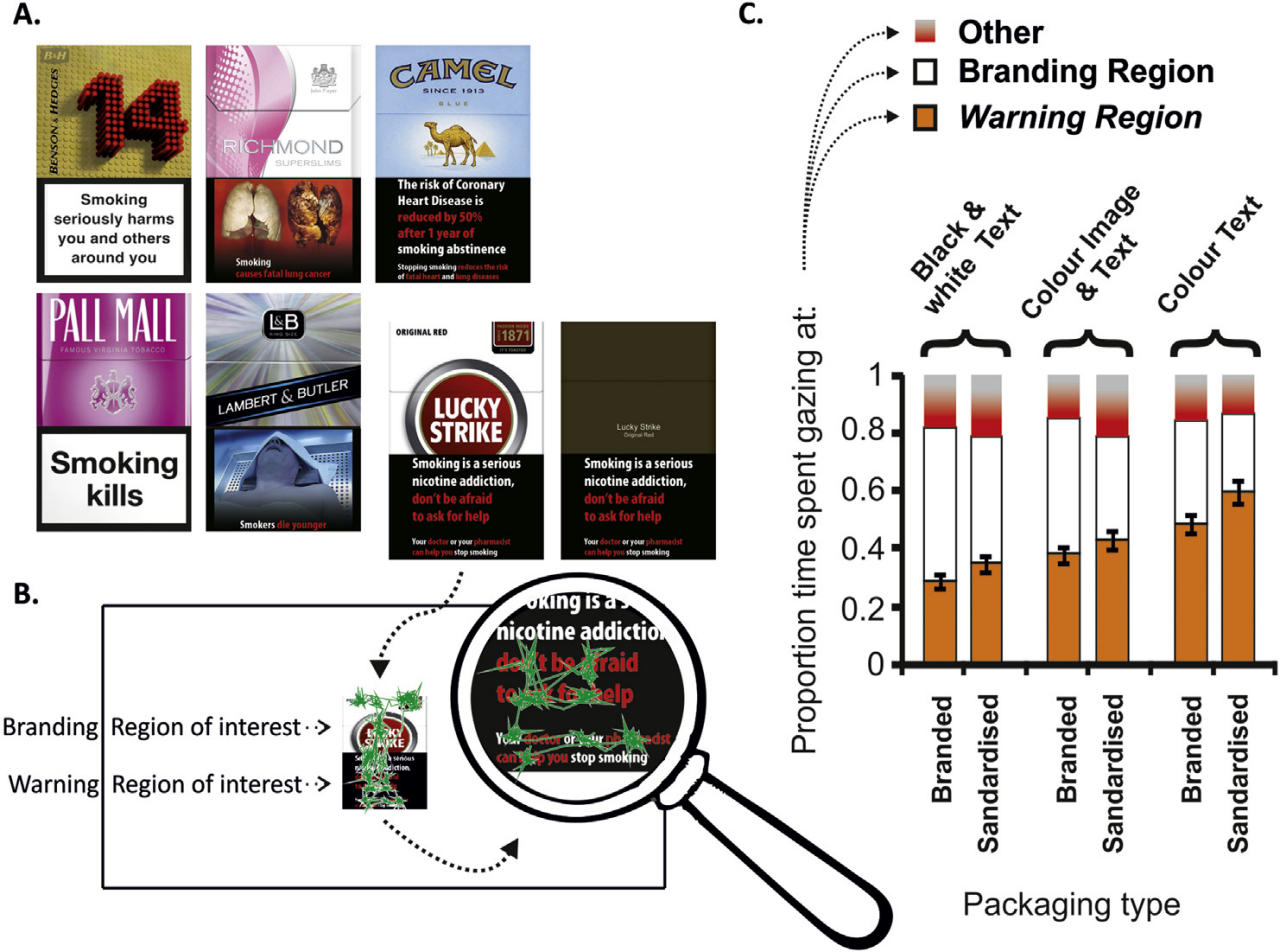
Stimuli and results. A. Illustration of the six brands and six warning labels used in the main experiment, divided into black & white text (left), colour image & text (centre) and colour text (right) warning-label conditions. The standardized version of one brand is also shown (bottom right). B. Screen shot from one example trial, with gaze position traced overlaid (in green). One section of the trace has been magnified for clarity. C. Mean proportion of viewing time spent fixating within the warning-label and branding regions of interest, or in ‘other’ locations (which includes samples where no eye position could be determined, e.g. blinks). Data are shown separately for the six experimental conditions. Error bars show standard error of the mean, and relate to the proportion of time spent fixating warning labels, our primary outcome measure.

The two black & white text warnings used were those currently employed on the front of cigarette packets in the UK: ‘Smoking Kills’ and ‘Smoking seriously harms you and others around you.’ The design implied matching these two black & white warnings with the same number of colour text and colour image & text warnings. Given that there are 15 colour health warnings currently in use on the back of cigarette packs in the United Kingdom (four colour text and 11 colour image & text), two colour text and two colour image & text warnings were selected based on a pilot study (see Fig. 1A). In the pilot study, 11 never-smoking participants completed the same procedure as in the main experiment, but with different stimuli. The fifteen current back-of-pack warnings were presented individually on screen, on both a standardized and branded version of the same cigarette pack (Marlboro), making a total of 30 images presented in random order. All 15 warnings evoked similar proportions of time spent gazing at the warning region of the pack (range = 0.54–0.67). The two colour text warnings and the two colour image & text warnings that gained the highest and lowest average proportions of viewing times within their category were selected for use alongside the two black & white text-only warnings, in order to capture the full range of current health warnings.

In the main experiment, each participant saw the selected six warnings twice each, once on a branded pack and once on the standardized version of that same pack. To counter any associations between particular brands and particular warnings, a Latin square was used to generate six different possible pairings of the six brands with the six warning labels. Then participants were rotated through these pairings in counterbalanced sets of six, thus ensuring that each warning appeared equally often with each brand across the full sample of participants.

### Analysis

Data for each participant (proportion of gaze time spent in the warning label region of interest in each condition) were copied to SPSS Statistics Version 21 (IBM, Chicago, USA) in order to assess group trends, which were analysed with factorial (2 × 3) analysis of variance (ANOVA) using the general linear model repeated-measures routine. The ANOVA tested the main effects of packaging style and warning type, and the interaction between them (to assess whether the effect of standardized packaging on gaze time varied significantly for the three different kinds of warning). Greenhouse-Geisser corrections were applied for violations of sphericity (i.e. heterogeneity in the variance of difference scores). Although it is not standard practice to break down factorial designs further in the absence of a significant interaction, the authors felt it was worthwhile to also assess statistical significance for each category of warning label when considered alone, and did so via *t*-tests. The analysis was exactly as planned at the time of study design, except that further investigation of any effects by demographic subgroups was not possible due to the homogeneity of the final sample.

## Results

Fig. 1B shows an example eye trace recorded in one trial for one participant and indicates the two regions of interest (warning region and brand region). Fig. 1C shows the mean proportion of time across all participants spent fixating within each region of interest for the two types of pack design and for each category of warning label. The primary outcome measure, the proportion of gaze time spent within the warning label region, is shown in orange.

Attention towards all categories of warnings increased when they were presented on standardized packs compared to branded packs (*F*(1,29) = 26.9, *p* < 0.001, partial *η*^2^ = 0.481). There were also clear differences in accumulated gaze time between the three types of health warnings, with greater gaze times for colour text compared to colour image & text, and for colour image & text compared to black & white text (*F*(2,58) = 52.6, *p* < 0.001, partial *η*^2^ = 0.645). Both of these main effects were highly significant, but there was no interaction between pack type and warning type (*F*(2,58) = 1.25, *p* = 0.295, partial *η*^2^ = 0.041). *Posthoc t*-tests (uncorrected) revealed significant differences in warning-label gaze times between standardized and branded packs for black & white text warnings (*t*_29_ = 2.14, two-tailed *p* = 0.041) and colour text warnings (*t*_29_ = 4.52, *p* < 0.001), but not for colour images & text warnings (*t*_29_ = 1.92, *p* = 0.065). It should be noted, however, that given the *a priori* expectation that standardized packaging would increase attention to warning labels, these comparisons might reasonably have been assessed as one-tailed tests, in which case all three reached conventional levels of significance (*p* < 0.05).

## Discussion

Previous research has suggested that standardized packaging has a positive effect in directing visual attention towards health warnings.^7,11^ This study aimed to extend these findings by varying the category of warnings alongside the packaging style in a factorial experiment. The findings demonstrate that, compared with branded packaging, standardized packaging significantly increases the time spent attending to health warnings on cigarette packets in a population of young adult never-smokers. Visual attention to warnings increased for all types of warning (colour text, colour image and text, and black & white text) when presented in the context of standardized packaging. There was no interaction between warning type and packaging style to suggest that the effect of standardized packaging varies depending on the kind of warning presented on the pack.

The finding in this study supports previous eye-tracking research demonstrating an increase in visual attention to health warnings in the absence of manufacturer branding in both adults and adolescents.^7,11^ Whereas those studies demonstrated an effect using colour image & text warnings, the results here extend these findings by also identifying significant effects for black & white text warnings and colour text-only warnings. Previously, effects of standardized packaging were found for adults (mean age 23) with <100 lifetime cigarettes smoked (i.e. a group very similar to our sample) and also for adolescents who experimented but hadn't smoked for at least a week.^7,11^ They were not found for adolescents who had never tried a cigarette, but this group spent a lot of time looking at health warnings regardless of packaging, which may have made them relatively insensitive to the packaging manipulation (perhaps reflecting their more naive interest in these warnings).

Compared to previous eye-tracking studies on this topic, a different type of task (asking participants to rate the appeal of each package rather than memorize packages) was introduced here. This change is relevant because strategic demands (i.e. the goals observers are trying to achieve) have long been known to affect eye movement patterns.^18^ In the task used here, there was actually no investigator-induced motivation to look at the health warnings at all, which more closely imitates real life situations. It is possible, however, that an instruction to rate ‘appeal’ could have enhanced the tendency to seek out areas of high image contrast (where relevant information might be expected to be found). A shorter display time relative to past research (5 compared to 10 s) was also used but a clear effect of standardized packaging was still obtained. Considered together with previous findings, these results suggest that the specific task is not a critical factor for generating a standardized-packaging-related enhancement of attention to health warnings. This increases the ecological validity of the data from eye-tracking experiments when considered collectively. Similarly, the specific choice of stimuli (e.g. the image display size) and the particular analysis method (accrued gaze time here vs number of saccades into a region of interest in previous full reports) do not appear to have been critical in generating packaging effects.

Given the physiology of the visual system, focussing upon warnings for as long as possible is a fundamental requirement to allow them to convey their health-related information. Other studies, using a variety of methods including both eye-tracking and attitude surveys, have provided some guidance about the effectiveness of different kinds of warnings, without considering their interaction with standardized vs branded packaging.^19–21^ Other research has also provided evidence about how the health beliefs and behaviour of consumers and non-consumers are affected once they have been successfully exposed to warnings. Current warning models suggest that, in addition to being seen, an effective health warning should aim to engage with a recipient's cognitive capacities, beliefs, attitudes, and motivation.^22^ However, none of this is possible unless the warning is seen for a sufficient period in the first place. This study's results suggest that standardized packaging will assist with this goal.

A minimum sample size was selected based on previous eye-tracking studies, with the proviso that additional participants would be tested if the target was reached before the predefined data collection end date. In fact, the minimum sample requirement was slightly exceeded. Importantly, there was no attempt to analyse the data prior to the end of the data collection period and thus increase the risk of Type 1 error. A relatively homogeneous sample was tested, mainly young female undergraduates. Although the age range of the sample is relevant for preventing smoking uptake, it would be beneficial to test for comparable results in other demographic groups to broaden the generalizability of the findings. It is also worth noting that standardized packs are a relatively new innovation, and it is possible that the novelty of the stimulus influenced patterns of visual gaze in this experiment. Future studies would benefit from exploring the longevity of these findings by testing participants again sometime after initial exposure. The findings in this study nonetheless add to the body of evidence in favour of standardized packaging, in a field marked by the relative paucity of objective behavioural results.^5^ Of course, any laboratory study faces questions regarding its applicability to real-world exposure. The authors look forward to more evidence emerging from the natural experiment of Australia's adoption of standardized packaging.

## Conclusion

This study demonstrates that standardized cigarette packaging affects the distribution of visual attention: an objective behavioural measure. Standardized packaging was found to increase attention towards the health warning region of cigarette packs. Previous findings were extended by showing that this occurred largely regardless of the type of warning employed on the pack. The results of this study have clear implications for regulations on tobacco packaging, currently under development in the UK. Standardized packaging has the potential to reduce the number of young people who start and subsequently become addicted to smoking each year, via the influence of health warnings. In concert with previous eye-tracking studies, the results suggest that standardized packaging would increase the salience, and thus the impact, of text and pictorial health warnings on non-smokers.

## Author statements

### Ethical approval

Ethical approval (dated 26.03.13) was granted (following light-touch procedures designed for minimal-risk social-scientific research with normal adult volunteers) by the Research Ethics Committee of the Department of Psychology, City University London. All participants provided informed consent.

### Funding

No specific funding was obtained for this study. KLM is funded by the Wellcome Trust (grant number 09401). KY is funded by the BBSRC (Grant Ref: BB/K01479X/1). These funders had no role in study design, the collection, analysis, and interpretation of data, the writing of the report, or the decision to submit the article for publication.

### Competing interests

All authors have completed the Unified Competing Interest form at www.icmje.org/coi_disclosure.pdf (available on request from the corresponding author) and declare no competing interests.
